# First evidence of overlaps between HIV-Associated Dementia (HAD) and non-viral neurodegenerative diseases: proteomic analysis of the frontal cortex from HIV+ patients with and without dementia

**DOI:** 10.1186/1750-1326-5-27

**Published:** 2010-06-24

**Authors:** Li Zhou, Eve Diefenbach, Ben Crossett, Sieu L Tran, Thomas Ng, Helen Rizos, Rejane Rua, Bin Wang, Amit Kapur, Kaushal Gandhi, Bruce J Brew, Nitin K Saksena

**Affiliations:** 1 Center for Virus Research, Westmead Millennium Institute, Westmead Hospital, The University of Sydney, Westmead, NSW 2145, Sydney, Australia; 2 Protein Production Facility, Westmead Millennium Institute, Westmead Hospital, The University of Sydney, Westmead, NSW 2145, Sydney, Australia; 3 School of Molecular and Microbial Biosciences, University of Sydney, NSW 2006, Australia; 4 Westmead Institute for Cancer Research, Westmead Millennium Institute, Westmead Hospital, The University of Sydney, Westmead, NSW 2145, Sydney, Australia; 5 Department of Anatomical Pathology, ICPMR, Westmead Hospital, Westmead, NSW 2145, Sydney, Australia; 6 The Australian Proteome Analysis Facility, Macquarie University, North Ryde, NSW 2109, Australia; 7 Microarray Facility, Westmead Millennium Institute, Westmead Hospital, The University of Sydney, Westmead, NSW 2145, Sydney, Australia; 8 Department of Neurology, St. Vincent's Hospital, Darlinghurst, Sydney, Australia; 9 Ecole Normale Superieure, 45 Rue Ulm, 75005 Paris, France

## Abstract

**Background:**

The pathogenesis of HIV-associated dementia (HAD) is poorly understood. To date, detailed proteomic fingerprinting directly from autopsied brain tissues of HAD and HIV non-dementia patients has not been performed.

**Result:**

Here, we have analyzed total proteins from the frontal cortex of 9 HAD and 5 HIV non-dementia patients. Using 2-Dimensional differential in-gel electrophoresis (2-DIGE) to analyze the brain tissue proteome, 76 differentially expressed proteins (p < 0.05; fold change>1.25) were identified between HAD and HIV non-dementia patients, of which 36 protein spots (based on 3D appearance of spots on the images) were chosen for the mass spectrometry analysis. The large majority of identified proteins were represented in the energy metabolic (mitochondria) and signal transduction pathways. Furthermore, over 90% of the protein candidates are common to both HAD and other non-viral neurodegenerative disease, such as Alzheimer's disease. The data was further validated using specific antibodies to 4 proteins (CA2, GS, CKMT and CRMP2) by western blot (WB) in the same samples used for 2D-DIGE, with additional confirmation by immunohistochemitsry (IHC) using frontal lobe tissue from different HAD and HIV+ non-dementia patients. The validation for all 4 antibodies by WB and IHC was in concordance with the DIGE results, lending further credence to the current findings.

**Conclusion:**

These results suggest not only convergent pathogenetic pathways for the two diseases but also the possibility of increased Alzheimer's disease (AD) susceptibility in HAD patients whose life expectancy has been significantly increased by highly active antiretroviral therapy.

## Background

HIV-1 associated dementia (HAD) is a common complication of HIV disease with a prevalence of at least 20% in advanced HIV infection in the pre-highly active antiretroviral therapy (HAART) era [1]. Even in patients taking HAART, milder forms of cognitive impairment remain common and functionally significant [2]. The reasons for the continued presence and development of HAD and its milder forms, despite effective HAART are not clear. Furthermore, due to the longevity of HIV patients after the advent of HAART, the prevalence of HAD has increased [3]. It has been hypothesized that Alzheimer's disease will significantly increase among elderly HIV-infected individuals [4]. Thus, there is the possibility of HIV initiating or facilitating a neurodegenerative process.

Various arrays and bioinformatic approaches have been utilized to explore the pathogenesis of HAD [5-9]. Based on the differentially expressed genes, many cellular processes, including T-cell receptor-mediated signaling, sub-cellular trafficking, transcriptional regulation, and a variety of cellular metabolic pathways have been identified. However, there are two issues with these studies. First, the transcriptomic gene expression has not been validated at the protein level. Second, several HAD pathogenesis proteome-based studies are only confined to cultured cells and cerebrospinal fluid (CSF) [10-13]. To date, protein changes directly in the native HIV-infected brain tissue have not been reported.

Therefore, in the present study, we employed 2D-DIGE, coupled with mass spectrometry, on the total protein extracts from the autopsied human frontal cortex tissue of HAD and HIV non-dementia patients to identify differentially expressed protein candidates between these two groups and to define the pathways and processes, which might be involved in the pathogenesis of HAD, along with any overlapping proteomic features between HAD and other neurodegenerative diseases, such as AD. Our study is unique in using the native brain tissues obtained from HIV+ individuals at autopsy for a detailed proteomic analysis.

## Results and Discussion

### Significant alteration of protein profiles in HAD brains as opposed to HIV non-demented brains

**Figure 1:**
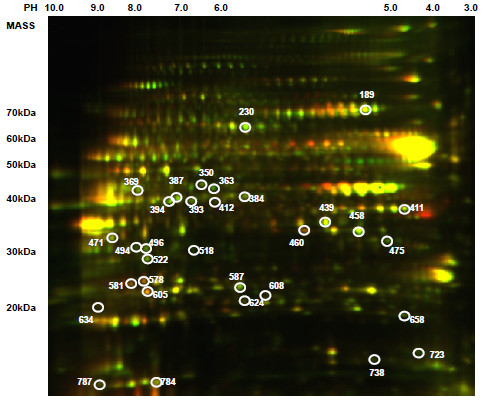
**Master gel from from 2D-DIGE experiment**. Master gel was chosen by DeCyder software automatically based on the spot numbers identified across all the gels. On this gel, one HAD sample (Cy5-labeled), one HIV non-dementia patient (Cy3-labeled) and an internal standard (Cy2-labeled) were included. First dimension, IEF pH 3 to 10 NL (right to left); second dimension, SDS (8-18%) polyacrylamide gel electrophoresis. White circles and numbers indicate identified proteins that are listed in Table 1.

In this study, we determined the significantly altered proteins between HAD and HIV non-dementia patients using 2D-DIGE coupled with mass spectrometry. We performed biological variance module analysis on 9 HAD and 5 HIV non-dementia patients. A total of 958 protein spots were detected on the master gel (893.14 ± 96.07 spots across all the individual gels), 76 of which were found to change significantly in HAD brains compared to HIV non-demented brains according to the criteria that a spot had to be present in at least 16 of the 21 images; the fold change had to be at least 1.25 with a *P *value less than 0.05. Figure 1 shows an image of the master gel. Among these 76 altered proteins, 36 were chosen to be identified by peptide mass fingerprinting, which was based on the 3-D appearance of spots on the images. Based on the data obtained from the MASCOT database, 2 proteins were found more than once and 3 gel spots contained more than one protein, making the total number of unique proteins to 31. Among them, 24 proteins increased significantly while the remaining 7 proteins decreased significantly. Table 1 shows the complete list of significantly altered proteins in the HAD brains when compared to HIV non-demented brains.

**Table 1:** Identified proteins and related pathways and neurological diseases summary

KEGG pathway	spot no	**Ac. No**.	Name	ratio	P value	Related Neurological disease
Glycolysis/Gluconeogenesis pathway	387	P09972	Fructose-bisphosphate aldolase C [Homo sapiens]	1.69	0.0009	Schizophrenia, bipolar disorder, and depression [100]
	471	P00338	L-lactate dehydrogenase A chain [Homo sapiens]	1.5	0.039	
	439	P07195	L-lactate dehydrogenase B chain (LDH) [Homo sapiens]	1.43	0.028	
	412	P14550	Alcohol dehydrogenase [NADP+] [Homo sapiens]	1.36	0.028	
	605	P60174	Triosephosphate isomerase [Homo sapiens]	-1.32	0.02	Neurodegeneration [101]
	581	Q53G35	Phosphoglycerate mutase 1 (Brain) variant (Fragment) [Homo sapiens]	-1.6	0.043	AD [102]

Oxidative phosphorylation pathway	518	B3KP20	cDNA FLJ30970 fis, clone HEART2000444, highly similar to Homo sapiens phospholysine phosphohistidine inorganic pyrophosphate phosphatase (LHPP), mRNA [Homo sapiens]	1.57	0.043	AD [85]
	522	P36543	V-type proton ATPase subunit E 1 [Homo sapiens]	1.55	0.0068	
	658	O75947-2	(ATP5H)Isoform 2 of O75947. [Homo sapiens]	1.48	0.015	
	587	P47985	Cytochrome b-c1 complex subunit Rieske, mitochondrial [Homo sapiens]	1.38	0.013	
	634	O96000	NADH dehydrogenase (ubiquinone) 1 beta subcomplex subunit 10 [Homo sapiens]	-1.42	0.0059	

Nitrogen metabolism pathway	363	P15104	Glutamine synthetase [Homo sapiens]	1.85	0.0007	AD [20]
	578	P00918	Carbonic anhydrase 2 [Homo sapiens]	-3.11	0.009	Mental retardation, AD [103,104]

Arachidonic acid metabolism pathway	494	P16152	Carbonyl r Carbonyl reductase [NADPH] 1 [Homo sapiens]	2	0.014	AD [105]

Purine metabolism pathway	787	P22392	Nucleoside diphosphate kinase B [Homo sapiens]	-1.33	0.0061	DS, AD [106]

Arginine and proline metabolism pathway	369	P12532	Creatine kinase, ubiquitous mitochondrial [Homo sapiens]	1.47	0.0006	Alzheimer's and Pick's Disease [17]

Glutathione metabolism pathway	624	P09211	Glutathione S-transferase P [Homo sapiens]	-1.64	0.024	Parkinson's disease, AD [107,108]

MAPK signalling pathway	738	P16949	Stathmin [Homo sapiens]	1.48	0.031	DSand AD [29,109]
	608	P62993	Growth factor receptor-bound protein 2 [Homo sapiens]	1.29	0.04	AD [110]

Calcium signalling pathway	496	B4DKM5	cDNA FLJ60120, highly similar to Voltage-dependent anion-selective channel protein 2 [Homo sapiens]*	1.57	0.021	
	411	P50148	Guanine nucleotide-binding Protein G(o) subunit alpha [Homo sapiens]	1.36	0.026	Familial Alzheimer's disease [49]

Axon guidance pathway	230	Q16555	Dihydropyrimidinase-related protein 2 [Homo sapiens]	1.57	0.025	AD [14]

Parkinson's disease pathway	384	Q7KYV2	H5 [Homo sapiens]*	1.37	0.035	Autosomal-recessive juvenile parkinsonism [111]

Antigen processing and presentation pathway	189	P11142	Heat shock cognate 71 kDa protein [Homo sapiens]	1.39	0.022	AD [59]

N/A	393	O00154	(ACOT7)Isoform 6 of O00154. [Homo sapiens]	1.64	0.0018	
	394	Q2TU84	Aspartate aminotransferase [Homo sapiens]	1.51	0.0048	
	
	350	P49411	Elongation factor Tu, mitochondrial [Homo sapiens]	1.35	0.008	Infantile Encephalopathy [24]
	723	P61601	Neurocalcin-delta [Homo sapiens]*	1.57	0.043	AD [53]
	
	475	B4DGP9	cDNA FLJ54102, highly similar to Beta-soluble NSF attachment protein [Homo sapiens]	1.53	0.033	
	
	458	P62879	Guanine nucleotide-binding protein G(I)/G(S)/G(T) subunit beta-2 [Homo sapiens]	1.73	0.0089	AD [46]
	
	784	A8MVL5	Putative uncharacterized protein PRDX5 [Homo sapiens]	-1.88	0.032	AD and parkinson [112,113]

Note: Most proteins in this table are involved in gene-ontology metabolic process except those proteins marked by *.

It is interesting to note that more than 90% of the proteins identified in the current study have been reported previously in relation to AD or other neurological diseases (Table 1), thereby lending further credence to our observations. Among them, 9 of 31 proteins have already been reported to interact with HIV directly or indirectly. According to their molecular functions, these 9 proteins can be categorized into 4 groups: proteins involved in metabolic pathways/processes; proteins involved in signal transduction pathways/processes; and antigen presenting protein. In the following section, we will discuss these nine proteins by their functions and three other functionally similar proteins, which have neither been reported previously in HIV infection nor in the context of other neurological diseases.

#### a. Proteins involved in metabolic pathways/processes

In the current study, we have found both creatine kinase (CK; EC 2.7.3.2) and glutamine synthetase (GS; EC 6.3.1.2) significantly increased in HAD brains in comparison to HIV non-demented brains. They are involved in energy related metabolism (arginine and proline metabolism and nitrogen metabolism, respectively). Furthermore, they are two of the three major specifically oxidized proteins in AD brains [14]. CK plays an important role in facilitating energy transfer within cells with high energy flux or requirements by catalyzing the reversible transfer of a phosphoryl group between adenosine-5'-triphosphate (ATP) and creatine. Cytoplasmic brain CK (BB form) and ubiquitous mitochondrial CK (uMtCK), among the four isoforms of CK, have been reported co-expressed [15] at various levels throughout the entire brain and serve as an efficient energy buffering and shuttle system in the brain [16]. For uMtCK, the change in AD brains is not significant although a sharp decrease of its activity has been reported [17]. Our results on the HAD brain proteome suggest that HIV can manipulate the energy production or transfer for its own use by altering uMtCK expression since HIV-1 Tat peptide can fuse with human brain CK, and this fusion can increase CK activity after being transduced into PC12 cells [18]. GS is a ubiquitous enzyme, which plays an important role in recycling the glutamate. It is mainly localized in astroglial cells. It has been used as a biomarker of oxidative stress [19] and potential diagnostic marker of AD [20]. Its presence is enhanced in neurological diseases associated with reactive astrogliosis [21]. Previous *in vitro *studies have also reported a positive correlation of GS with HIV replication [22] or the concentration of HIV glycoprotein 120 [23]. Our findings provide *in vivo *evidence that HIV infection can alter GS expression in human brain, thereby impairing the glutamine/glutamate cycle.

Further, in this context, it is important to mention that we identified another protein, elongation factor Tu, mitochondrial (EF-Tu), which plays a vital role in energy-related cellular metabolic processes and can interact with HIV. It is involved in the mitochondrial protein translation and its mutations are associated with combined oxidative phosphorylation deficiency, which can lead to fatal encephalopathy [24]. Previous studies have shown that EF1a, one of its family members, can interact with the entire HIV-1 Gag polyprotein [25]. Therefore, the increase of EF-Tu in HAD brains, observed in the current study, raises the possibility that HIV-host interaction might partly contribute to the abnormal oxidative phosphorylation. Thus, HIV might be able to trigger oxidative stress in the HAD brains, which is a vital step in the development of neurodegeneration. Our findings also concur with a previous *in vitro *study that HIV-1 infection does induce the generation of reactive oxygen species (ROS) [26]. Further *in vivo *studies on enzyme activity and protein modifications are needed in the context of neurological manifestations of HIV disease.

#### b. Proteins involved in signal transduction pathways/processes

Two important proteins, stathmin (STMN1) and growth factor receptor-bound protein2 (GRB2), identified in our study, are involved in mitogen activated protein kinase (MAPK) signalling pathways, which are known to play multiple roles in HIV disease and NK effector functions [27,28]. Up-regulation of STMN1 in HAD brains was found in our study, whereas a decrease of STMN1 had been previously reported in AD and DS brains [29]. STMN1 belongs to a class of regulatory proteins related to microtubule dynamics [30] and is vital for cellular processes, including intracellular transport, maintenance of cell shape and cell polarity [31]. It is widely distributed in the neuronal cell body, dendrites, axons, and growth cones [32] and is involved in the destabilization of microtubule, which is essential for axon and dendrite differentiation, growth, and maintenance and even the generation of neuronal size, shape, and compartmentalization [33]. In addition, Nishi et al. [34] have demonstrated that STMN1 expression can inhibit enhancement of HIV-1 particle production by suppressing cytokine signaling-1. Our results indicate that alteration of STMN1 might be one of the positive host responses upon HIV infection at the protein level, which can inhibit the virion assembly and production.

GRB2 is an adaptor protein involved in signal transduction/cell communication and T-cell activation [35]. GRB2 coupled to Son-of-sevenless-1 (SOS-1), which can activate the RAS/MAPK pathway [36], and form one of the primary components of signal transduction cascade [37]. In neurodegenerative processes it directly interacts with amyloid precursor protein (APP) and presenilin (PS1), which play an important role in the onset of AD [38]. Moreover, increased levels of Grb3-3, an isoform of Grb2, were seen in PBMCs from HIV-1 infected subjects [39]. In the current study, a mild increase of Grb-2 protein was seen in HAD brains, which complements previous studies on AD brains. Its increase in HAD brains is probably triggered by HIV-1 since Grb3-3 can be induced by HIV Tat and Nef proteins independently [40]. In the context of HAD, it might be involved in the MAPK activation and possibly tau hyper-phosphoylation, which needs to be elucidated in future.

The guanine nucleotide-binding proteins (G proteins) families also play a crucial role in signal transduction. They function as molecular switches between extracellular events and intracellular effectors. Through the coupling between their receptors and several heterotrimeric G-proteins in NK cells, the C, CC, CXC and CX3C chemokines can activate NK cells and induce intracellular signalling pathways in the NK cells [41,42]. In the present study, we found a significant up-regulation in both G_o _subunit alpha and beta-2. Our results are consistent with previous *in vitro *array studies [7]. It might indicate a stronger host antiviral response by activating the NK cells and then death of the infected host cells. In this context, it is important to reiterate that the CC chemokine RANTES, MIP-1b and the CXC chemokine SDF-1a, which signal through M-tropic HIV-1 co-receptors CCR5 and CCR3 and T-tropic HIV-1 co-receptor CXCR4 respectively, are activated by G proteins [43,44]. In non-viral neuro-degeneration, G-protein can also couple with two of the three causative gene products of familial Alzheimer's disease, APP [45] and PS1 [46]. Data (reviewed by Cowburn [47]) have suggested that the neurochemical pathology of AD includes severe disruption of the neurotransmitter receptor/G-protein mediated phosphatidylinositol hydrolysis and adenylyl cyclase signal transduction pathways. β_2 _subunit and β γ complex of G-proteins have been reported to participate in chemokine-induced NK cell chemotaxis [48] and mediate apoptosis [49], respectively. In the context of HIV, further studies are needed to explore the mechanism of how HIV interferes with G proteins expression and utilizes G proteins to manipulate the signalling pathways for its survival.

Another important protein interacting with HIV identified in this study is neurocalcin, which is a Ca^2+^-binding protein distributed abundantly in the central nervous system and has been reported to play a role in neuronal signalling [50]. So far, at least 6 isoforms of neurocalcin have been identified. Neurocalcin δ is expressed mainly in glial cells [51]. It has been reported that one of the target proteins of neurocalcin δ is S100β [51], which has been shown to be up-regulated in HIV infection and very important to HAD neuropathogenesis [52]. To the author's knowledge, neurocalcin δ has been only reported to be site down-regulated in the temporal lobe of AD brains [53,54] without comparison to control brain. In our study, we found an up-regulation of neurocalcin in the frontal cortex of HAD brains and our results suggest that neurocalcin δ and S100β complex might participate in the pathogenesis of HAD by inducing gliosis, growth of dystrophic neurites, and calcium-mediated neuronal cell loss.

#### c. Antigen presenting and other proteins

Heat shock cognate 71 kDa protein (HSC-71) was mildly but significantly increased in the HAD brains. HSC-71 is the constitutively expressed member of the heat shock protein 70 (Hsp70) family and has 85% homology with Hsp70. Hsp70 protein level can increase due to HIV infection or oxidative stress [55,56]. Furthermore, Hsp70 can trigger an increased immune response by incorporating into the membrane of HIV virions and prevent HIV induced astrocytes apoptosis [57]. In the context of neurodegeneration, HSC71 plays more important roles. For instance, it can interact with the cytoplasmic domain of APP in the presence of proteasome inhibitors. This indicates that HSC-71 might participate in proteasome structural maintenance and mis-folded protein conformational recognition [58]. Although HSC-71 has been reported to have an insignificant down-regulation in the AD brains [59], it appears more oxidatively modified and possibly glycosylated in the AD brains [14,60]. Taking these observations in the context of our study, the increase in the expression of HSC-71 could be a consequence of HIV infection or increased oxidative stress seen during HIV infection [61].

Worthy of noting is that we have found three functionally similar proteins (ACOT7, FLJ54102 and FLJ60120), which have neither been reported previously in HIV infection nor in the context of other neurological diseases. ACOT7 or Acyl-CoA thioesterases (EC 3.1.2.2.) are enzymes that catalyze the hydrolysis of CoA esters of various molecules to the free acid plus coenzyme A (CoA) [62], which differentiates them from long-chain acyl-CoA synthetases because long-chain acyl-CoA synthetases ligate fatty acids to CoA, to produce the CoA ester [63]. They are implicated in the regulation of intracellular levels of CoA esters, the corresponding free acid, CoASH and cellular processes involving these compounds. ACOT8 under recently revised nomenclature was identified as hACTEIII [64] and hTE [65], which can interact with and activate the HIV-1 Nef protein. So far, only long chain acyl-CoA synthetases have been reported to be related to neurodegenerative disease [66]. Interestingly, we found up-regulation of the ACOT7 protein uniquely in the HAD brains implying its likely interaction with HIV.

The second protein is cDNA FLJ54102, a highly similar protein to beta-soluble N-ethylmaleimide-sensitive factor (NSF) attachment protein. We observed an up-regulation of FLJ54102 in the HAD brains. Soluble NSF attachment proteins (SNAPs) are highly conserved proteins, which are involved in intracellular membrane fusion and vesicular trafficking. There are three individual isoforms of SNAPs: α, β, and γ [67]. Among them, β-SNAP is brain specific and it has been reported that maximal levels of its expression are in the hippocampus [68]. Schiavo [69] showed that β-SNAP, NSF, SNAP receptor, and the calcium-binding protein synaptotagmin (SYT) assemble cooperatively to form a docking and fusion complex. HIV Tat has been reported to be able to fuse with NSF and inhibit the extrocytosis [70], while SNAP receptor has been reported to be involved in fusion events of endosomal Gag-RNA complexes with the plasma membrane to generate virions through an endosome-dependent route [71]. The expression of SNAPs has also been shown to be significantly reduced in the AD and DS brains [72].

The third is cDNA FLJ60120, highly similar to voltage-dependent anion-selective channel protein (VDAC) 2. VDAC is a mitochondrial outer-membrane protein, which plays an important role in the pore formation and cytochrome c release [73]. It can regulate cell death by binding to B-cell CLL/lymphoma 2 (Bcl-2) family pro- or anti- apoptotic protein [74,75]. It can also regulate the mitochondrial function by controlling metabolite fluxes through the mitochondrial membrane [76,77]. It can impact on glucose metabolism by directly binding to glycolytic enzymes [74]. More importantly, it has been reported to be involved in neurodegenerative disorders and mental retardation [78-81]. By binding VDAC, HIV Vpr can induce apoptosis through a direct effect on the mitochondrial permeability transition pore complex (PTPC) [82]. Three of human VDACs have been cloned and termed, VDAC1, VDAC2 and VDAC3. VDAC2 has been shown to over-express in the AD brains [74]. Our findings on HAD brains suggest possible association with apoptosis and the synaptic loss in HAD brains due to the impairment of energy pathways during HIV infection.

Apart from these proteins discussed at above, other identified proteins in the current study so far haven't been reported to be related to HIV infections. However, some proteins, especially carbonic anhydrase 2 (CA2) and carbonyl reductase [NADPH] 1, changed dramatically in the HAD brains (Table 1). Moreover, both of them have been reported to change in AD brains and are involved in AD pathogenesis. Thus, our results might indicate that HIV is able to alter those candidate proteins that directly or indirectly affect neurological functions. This sheds light on some novel candidate host proteins that possibly interact with HIV and modulate neuropathogenesis.

### Pathway and network analysis

For annotation and pathway analysis of these identified proteins, Metacore and DAVID were used. The 31 identified differentially expressed proteins were transferred into the official gene symbol ID code based on their corresponding Swiss-prot database accession numbers. There were 19 statistically significant locations (FDR<0.05, p < 0.01), the details of which are shown in the Additional file 1. Among them, the mitochondrion, cytoplasm, cytoplasmic part, cytosol and mitochondrial inner membrane rank the top 5 according to the statistical significance. Functionally, most of them are enzymes, while several transporting proteins and generic binding protein were also observed (see Additional file 2).

**Figure 2:**
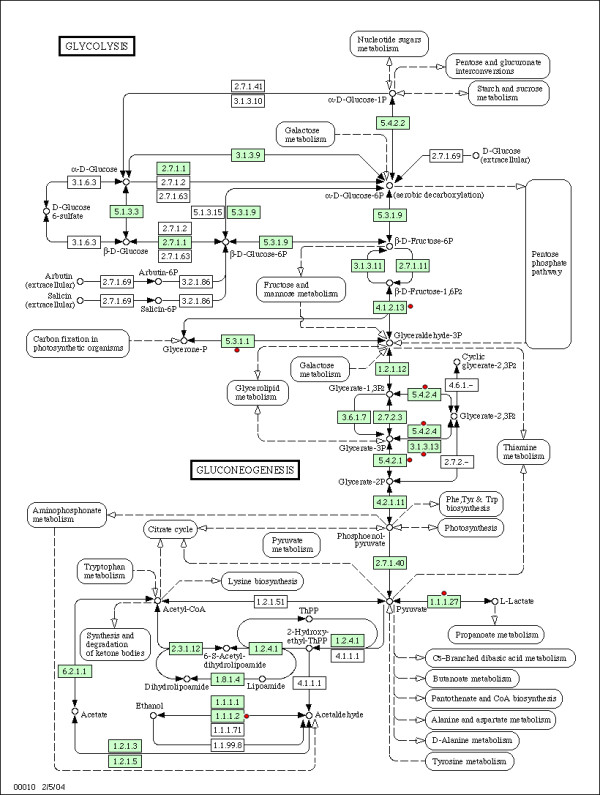
**Illustration of altered components of HAD frontal cortex in the Glycolysis/Gluconeogenesis pathway**. Figure 2 depict the classical Glycolysis/Gluconeogenesis pathway obtained from the KEGG pathway database http://www.genome.jp/kegg/. The genes, whose corresponding proteins have been found to differentially change in the current study, are highlighted in red and are denoted by dots. The protein details are listed in Table 1.

**Figure 3:**
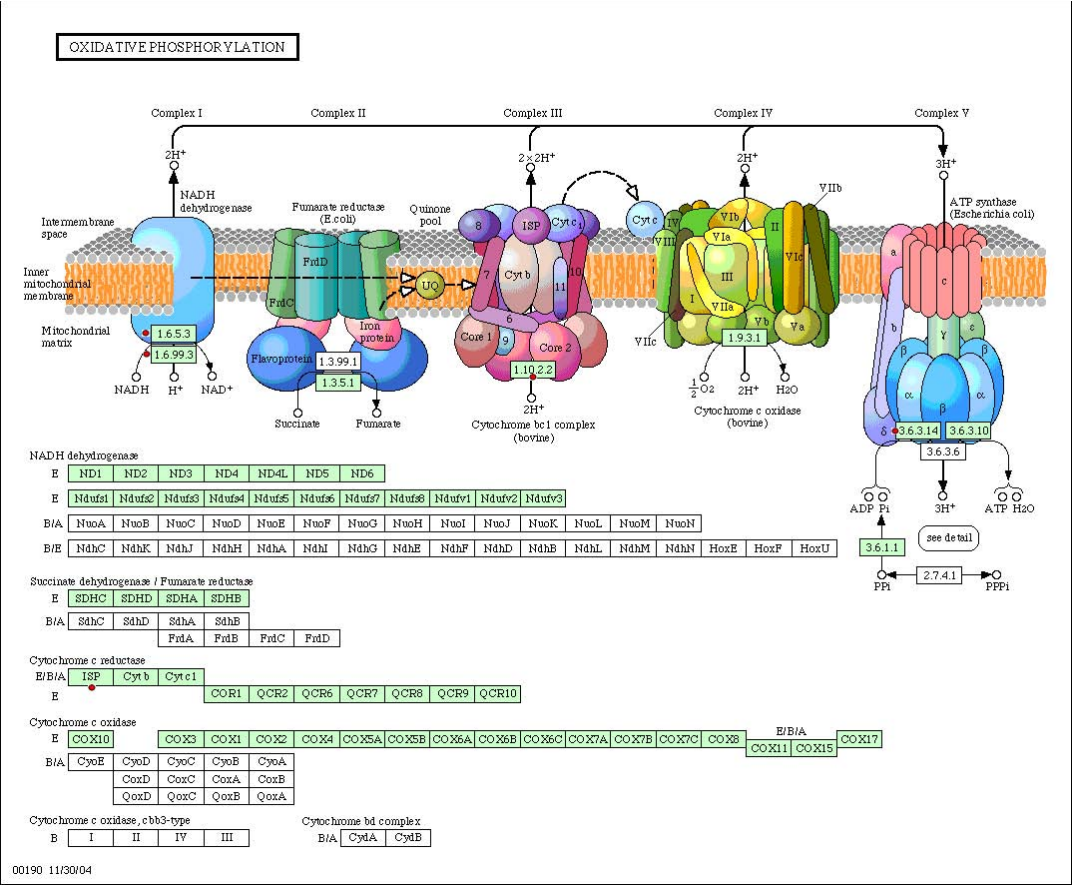
**Illustration of altered components of HAD frontal cortex in the Oxidative phosphorylation pathway**. Figure 3 depict the classical Oxidative phosphorylation pathway obtained from the KEGG pathway database. The genes, whose corresponding proteins have been found changed in the current study, are highlighted in red and are denoted by dots. The protein details are listed in Table 1.

**Figure 4:**
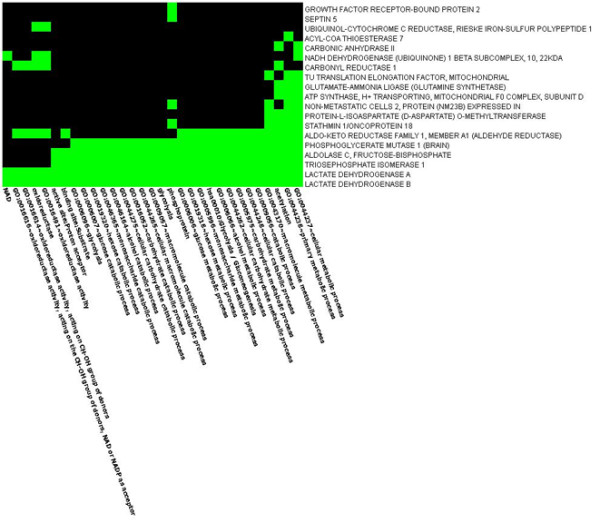
**Heatmap showing evidence of protein enrichment in the Glycolysis/Gluconeogenesis pathway**. Figure 4 shows the heatmap depicting enrichment of proteins in the Glycolysis/Gluconeogenesis pathway. Rows signify enriched genes and the columns signify related processes within the pathway. Green cells indicate that the corresponding genes and terms are associated positively according to the literature, whereas the black cells indicate the association not yet been reported.

**Figure 5:**
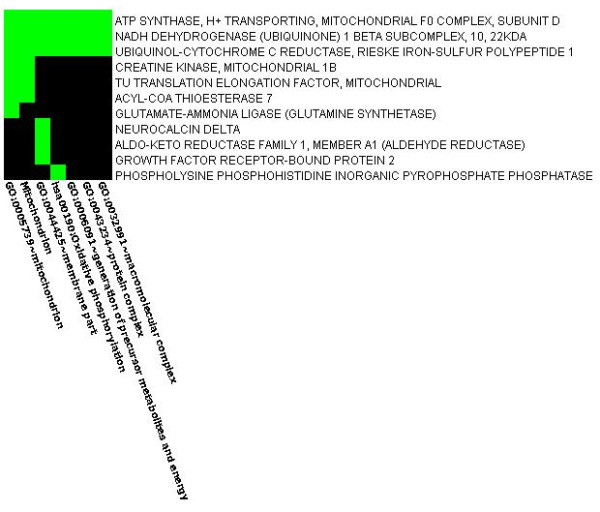
**Heatmap showing evidence of protein enrichment in the Oxidative phosphorylation pathway**. Figure 5 is the heatmap showing enrichment of proteins in the Oxidative phosphorylation pathway. Rows signify enriched genes and the columns signify related processes within the pathway. Green cells indicate that the corresponding genes and terms are associated positively according to the literature, whereas the black cells indicate the association not previously reported.

The pathway analysis showed that the Glycolysis/Gluconeogenesis and Oxidative phosphorylation KEGG pathways were highly enriched (enrichment score are 3.82 and 1.21, respectively) and statistically significant (p = 3.7e-6 and 1.6e-2, respectively). The key enzymes of Glycolysis pathway, TPI and PGAM1, were significantly down-regulated while other identified proteins within the Glycolysis pathway (ALDOC, AKR1A1, LDHA and LDHB) were up-regulated (Figure 2). In the Oxidative phosphorylation pathway, the proteins involved in complex III and complex IV (LHPP, ATP6V1E1, ATP5 H, UQCRFS1) were up-regulated, only NDUFB10, an important protein in complex I, was down-regulated (Figure 3). The association between enriched genes and related processes within these two pathways is shown in Figure 4 and Figure 5.

**Figure 6:**
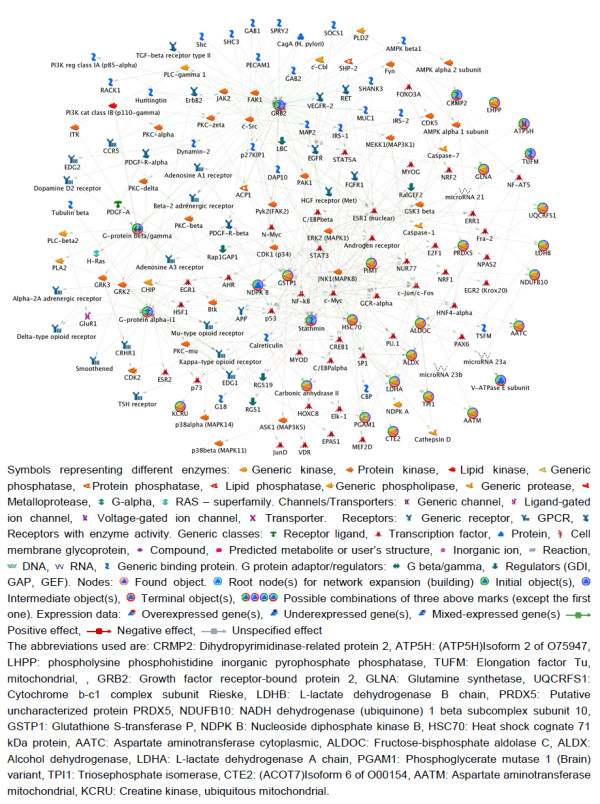
**GeneGo network**. Proteins identified in this study were uploaded to the Metacore software (GeneGo corp). The generated network shows significant involvement of proteins in the metabolic process. Interactions between proteins are denoted by lines. Green lines indicate activation, while the red lines indicate inhibition. Nodes are represented by distinct shapes and colors.

We next examined the gene-ontology biological process networks. There are 48 processes that are highly significant based on our data with FDR<0.005 and p < 0.0005 (FDR cut off value, stringently<0.05, normally<0.25), (see Additional file 3 for a complete list). The most significant one is the generation of precursor metabolites and energy process (p = 2.095e-9), which contains 11 identified proteins out of 31. The most involved is the metabolic process (p = 1.604e-5), which contains more than 90% of the identified proteins (Figure 6). The identified proteins are involved in diverse metabolic processes, including carbohydrate metabolism, energy metabolism, lipid metabolism, nucleotide metabolism and amino acid metabolism.

It is broadly accepted that metabolic pathways/processes are closely related to neurodegenerative diseases. It has been shown that the impairment of energy metabolism can exacerbate synaptic dysfunction, neuronal injury, which together may lead to neurodegenerative disorders [83]. The oxidative phosphorylation system (OXPHOS), using glucose as critical substrates, normally provides more than 95% of ATP used for cellular energy. Inefficient glycolysis plays a crucial role in the pathogenesis of AD [84]. Further, the mitochondrial genes in complex I of OXPHOS have been demonstrated to be significantly down-regulated in AD and Parkinson's brains [85,86]. In the context of HIV, it has also been shown that HIV-1 targets the energy generating system of the host cells by affecting mitochondrial DNA and protein [87]. HIV envelope glycoprotein gp120 can impair glucose metabolism *in vitro *and *in vivo *[88,89]. Furthermore, HIV-1 matrix protein, p17, can stimulate gluconeogenesis by inducing the expression of Fructose 1, 6 bisphosphatase, which can convert fructose-1, 6-bisphosphate to fructose 6-phosphate and produce NADPH [90]. Also, it has been reported that HIV can reduce the activity of complex I by down-regulating the expression of NDUFA6 at protein level [91].

Supporting these arguments, our pathway/process analysis has shown that most of the identified proteins in the current study were involved in metabolic pathways or processes. Figure 2 and Figure 3 shows the Glycolysis and Oxidative phosphorylation KEGG pathways. We have observed a significant decrease in the levels of two key enzymes of Glycolysis pathway (TPI and PGAM1) and complex I of Oxidative phosphorylation pathway (NDUFB10), while complex III and complex IV were up-regulated. These findings overlap with AD brains as well [85]. It is probably due to a great demand on energy production. In addition, it has been shown that HIV-1 infection does induce ROS, thereby leading to T cell death [26]. Our results provide a further explanation that HIV might influence the production of ROS through regulating the Oxidative phosphorylation pathway. Figure 6 shows the metabolic network, which includes identified proteins in the current study (marked with a circle), and the proteins within the metabolic network and closely interact with the identified proteins. All edges are supported by at least 1 reference from the literature. Collectively, this complex alteration of proteins provides strong support for abnormalities in metabolic pathways/processes during HAD in humans.

### The overlap between HAD and non-viral dementia

It is interesting that more than 90% of the proteins identified in the current study share an overlap with proteins reported from other neurodegenerative brains (Table 1). Although the alteration of these pathways/processes and proteins are possibly more generalized in neurodegenerative disorders rather than specifically abnormal in individual diseases, the critical overlap between HAD and non-viral neurodegenerative disorders, such as AD, is worthy of attention. Previous studies have shown that brain infections, such as herpes simplex, are related to the occurrence and development of AD process [92-94]. In addition, Brosseau et al. [95], have shown the first case of HIV-associated dementia with characteristics of Alzheimer's disease in a patient with AIDS, which supports the functional and neurophysiological relevance of the data shown in this study. Our findings at the proteomic level further raise the possibility that HIV might initiate or facilitate a neurodegenerative process. Our findings are consistent with previous reports at the genetic level [96]. However, until detailed proteomic fingerprinting is available from various virus-mediated neurodegenerative diseases (such as HSV encephalitis), it is difficult to rule out whether these overlaps at the protein level exist only between HAD and non-viral neurodegenerative diseases or human brain responds to different pathogens similarly. Nonetheless, our study is the first to provide this tantalizing evidence in favor of this major proteomic overlap between HAD and non-viral neurodegeneration, which in the future may clarify the involvement of any pathogenic etiology in non-viral neurodegenerative processes.

### Functional validation

**Figure 7:**
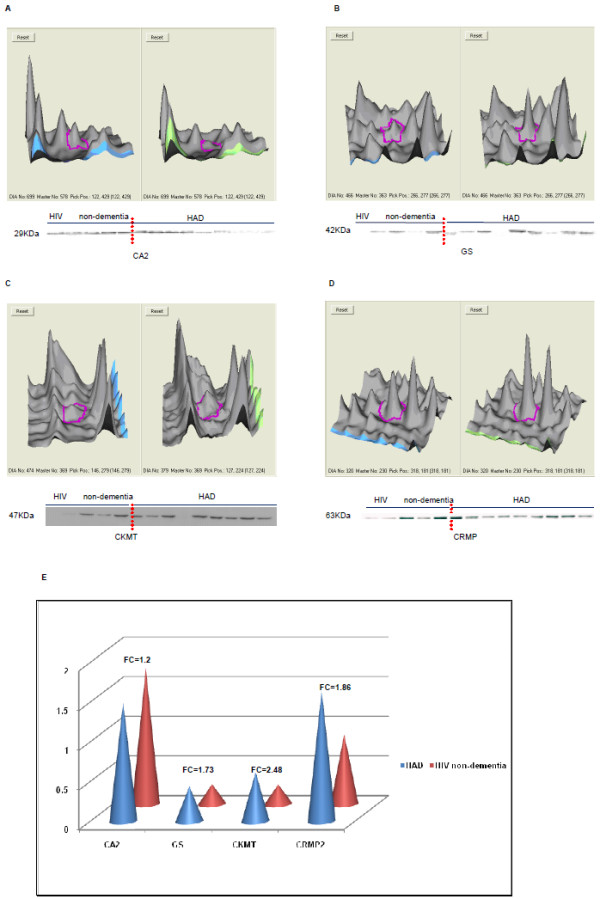
**3D DeCyder image and corresponding Western blot analysis for four representative proteins**. Each pair of protein spots (Cy3-and Cy5-labeled) in 3D views is shown together with the corresponding western blot analysis (A: CA2, B: GS, C: CKMT, D: CRMP2). The 3D peak of each protein was generated based on the pixel intensity versus pixel area, whereby the peak area correlated with the distribution of a given protein spot on the gel and then normalized by the standard (Cy-2-labeled). 3D images were obtained from DeCyder software. The western blot results correlated with the 2D-DIGE data. Sample orders in western blot analysis were the same as shown in Additional file 4, from right to left. Sample #2434 and #H0011db were absent in GS and CKMT proteins. Semi-quantitative western blot analysis (E) represented the relative protein level (standardized by Actin) in HAD and HIV non-dementia patients. Fold-change in values was labelled on the top of each paired comparison. The quantification analysis demonstrated the trend similar to the one observed in 2D-DIGE for HAD patients when compared to HIV non-dementia patients (p < 0.05).

Western blotting was performed to validate the 2D-DIGE data using the same samples for a subset of 4 proteins: carbonic anhydrase 2 (CA2), glutamine synthetase (GS), creatine kinase, ubiquitous mitochondrial (CKMT), and dihydropyrimidinase-related protein 2 (CRMP2). CA2 is down-regulated protein in the current proteomic study and its deficiency is closely related to other neurological disease, therefore it was chosen for validation by western blot. CA2 was recognized by Carbonic Anhydrase II antibody at size of 29 kDa (Figure 7A). It was expressed in both HAD and HIV non-dementia brains, but its relative abundance was slightly higher in HIV non-dementia patients as opposed to the dementia group (FC = 1.2, p < 0.05, Figure 7E). This is consistent with our IHC result. This is probably due to the high abundance of this protein, which can influence the fold-change accuracy. Moreover, this difference was more prominent when severe dementia and non-dementia patients were compared (FC = 6.7).

In contrast, the GS, CKMT, and CRMP2 were up-regulated proteins, which changed from mild (FC<1.5) to moderate (1.5<FC<2). Apart from their direct relevance in neurological disease, they were also chosen for validation because of their increase in fold-change in 2D-DIGE data. They were recognized by their specific antibodies at sizes 42, 47 and 63 kDa, respectively (Figures 7B, C and 7D). The western blot results of these three proteins followed the trend of 2D-DIGE data (p < 0.05) but not in numeracy (Figure 7E), with the exception of two samples where they were not recognized by the antibodies (sample #2434 and #H0011db with GS and CKMT). This is possible because the low sensitivity of western blot techniques/antibodies compared to 2D-DIGE/CyDye. Although some noticeable sample-to-sample variation within the same group was observed, no statistical relationship with dementia stage was found apart from some visual differences in relationship. Figure 7 shows the 3D DeCyder interpretation of all 4 proteins together with their corresponding western blot results.

**Figure 8:**
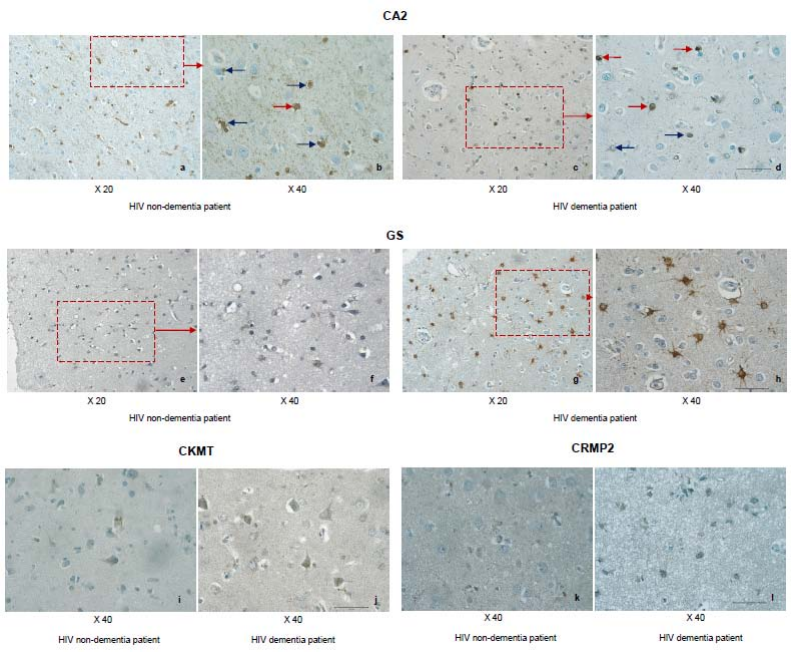
**Immunohistochemical staining of four representative proteins in HAD and HIV non-dementia patients in the frontal lobe**. Immunohistochemical evaluation of CA2 (a, b, c and d), GS (e, f, g and h), CKMT (i and j) and CRMP2 monoclonal antibodies (k and l) for staining the frontal lobe from HAD and HIV non-dementia patients. Relatively less CA2 staining, but more focal and neuronal staining was observed in HAD patient, as seen at different magnifications (a: x20, b: x40) compared to a more spread out astrocyte staining in HIV non-dementia patients (c: x20 and d: x40). The red arrows on b and d showed neuronal staining, while the blue arrows show astrocytic staining. Extensive GS staining was seen in HAD brain (g: x20 and h: x40), whereas in the HIV non-dementia patient the staining was significantly weaker (e: x20 and f: x40). The staining with CKMT and CRMP2 antibodies showed relative weaker signals due to low antigen levels. However, the clear differences are still noticeable for both antibodies. For CKMT, in HAD patient (j), there are strongly stained cells, along with several weakly stained cells around, whereas in HIV non-dementia patients (i), only very limited number of stained cells with only fewer weakly stained cells around. This contrast was stronger in CRMP2 staining (k and l) in comparison to CKMT.

Further, we also performed additional validation using immuno-histochemistry to confirm the results obtained from 2D-DIGE experiments and also western blots using the frontal lobe brain tissue sections derived from patients with and without HAD for the same 4 proteins used in WB analysis. The staining of three up-regulated proteins (GS, CKMT and CRMP2) was in concordance with the 2D-DIGE results. All three proteins stained for astrocytes, especially in the superficial cortex proximal to the leptomeninges. Protein GS stained on astrocytes in the white matter, whereas it was much weaker when compared to staining in the cortex. Considerably significant difference was observed between GS staining in HAD and HIV non-dementia brains. For CKMT, apart from astrocytes, it occasionally stained on scattered microglia. More prominent and positive staining for CKMT and CRMP2 was found in the HAD brain as opposed to HIV non-dementia brain. Possibly owing to the low-abundance of CKMT and CRMP2, these differences are not so prominent, but these results are fully consistent with the mild change of these two proteins in 2D-DIGE results. CA2 protein was found predominantly in relatively small sized neurons, while the bigger size neurons were negative for CA2 staining. The biological reason for which is not clear. In addition, it was weakly stained on some astrocytes as well. Compared to HIV non-dementia brain, the staining of CA2 in the HAD brain was very focal in some areas, while almost absent in others. In contrast, in HIV non-dementia brain, the staining was well spread implying that the CA2 expression could be pathology-specific, which need to be elucidated in future study. Overall, the CA2 staining was comparatively lesser in the HAD brain as opposed to non-HAD brain, which further confirmed the trend of 2D-DIGE, but not in numeracy. The reason for this could be the high abundance of this protein *in vivo*, which cannot accurately provide high-fold change comparison. Alternatively, the section from HAD patient is rich in CA2-related pathology confirmed by western blot results, which also showed the variation within group. Thus, a bigger sample size is needed to elucidate this hypothesis in future studies. Figure 8 shows immunohistochemical staining results for all 4 antibodies discussed in this section.

## Conclusion

Our study is the first demonstration of evidence showing overlapping proteins between viral (HIV) and non-viral neurodegenerative diseases. Although the majority of proteins identified in this study have been previously reported in relation to other neurodegenerative or psychiatric diseases, these findings do provide a strong foundation not only for understanding possible mechanisms of HAD, but also provide a much needed foundation for clarifying the possible involvement of a pathogenic etiology in non-viral neurodegenerative diseases. Furthermore, we also observed a significant involvement of Glycolysis and Oxidative phosphorylation, the two important energy related metabolic pathways, in the HAD brains, which has been fully demonstrated in the current study at the pathway level. Interestingly, the involvement of these pathways in HAD also coincides with their involvement in other neurodegenerative diseases, such as AD and PD.

## Materials and methods

### Patient details and brain tissue collection

Brain tissue samples were obtained from HIV-1-infected patients with or without HAD through the National Neuro-AIDS Tissue Consortium (NNTC, Request #R203) and the Westmead Hospital, Sydney, Australia (Reference No: 5465). Samples were collected at post-mortem, shipped frozen on dry ice and stored frozen at -70°C until use. Frontal cortex of male patients 9 with HAD and 5 without were used for this study due to its importance to motor impairment and involvement in AD [97,98]. The average age for HAD and non-HAD patients was 43.57 ± 14.77 and 50.2 ± 11.88, respectively, (P = 0.41). Clinical profiles of all patients are shown in Additional file 4. This study was conducted according to the principles expressed in the Declaration of Helsinki. Use of samples in this study was approved by Institutional Review Board and the Ethics Committee of the NNTC Allocations, the University of Sydney and the Westmead Hospital individually. The family members of the patients have given written, informed consent for the use of autopsied brain tissue. For the diagnostic criteria for HAD, the criteria defined by the American Academy of Neurology 1991 were used (American Academy of Neurology. Nomenclature and research case definitions for neurological manifestations of HIV type 1 infection 1: Report of a working group of AAN of neurology and AIDS task force, 1991).

### Sample preparation

In each case, 30 mg of frozen brain tissue was measured on dry ice and then transferred into a 2 mL Eppendorf tube. The tissues were lysed in 1 mL of 4°C lysis buffer (7 M urea, 2 M thiourea, 30 mM Tris, 4% CHAPS, 2% ASB, 1% Sigma protease inhibitor cocktail), vortexed for 30 seconds and cooled for 1 minute on ice (repeat 25 cycles), sonicated for 1 minute at 4°C and cooled for 1 minute on ice (repeat 3 cycles), and centrifuged for 50 minutes at 14000 rpm at 4°C. Protein concentrations were determined using the 2-D Quant kit (GE Healthcare), as per manufacturer's instructions.

### Protein labeling with CyDyes

Protein samples were labelled with CyDyes (GE Healthcare), as per manufacturer's instructions. 25 μg of total protein from each sample was mixed in an Eppendorf tube (Eppendorf, Düsseldorf, Germany) and labelled with Cy2 minimal dye, and 50 μg protein was taken from the mix and used as an internal standard on each gel for the subsequent 2D electrophoresis and image analysis. In parallel, 50 μg proteins of each sample were labelled with either Cy3 or Cy5, and the dyes scrambled within each group to avoid possible dye bias. The sample volumes were adjusted to 18 μL with labelling buffer (7 M urea, 2 M thiourea, 4% CHAPS, 30 mM Tris), followed by addition of 1 μL dye (working solution) to each individual sample. The samples were left on ice for 30 minutes in the dark, followed by adding 1 μL of 10 mmol/L lysine to stop the reaction.

### 2D Electrophoresis and image analysis

One sample from each of the CyDye groups was mixed together and adjusted to final concentrations of 1% DTT, 1% IPG buffer at a total volume of 350 μL with lysis buffer (7 M urea, 2 M thiourea, 4% CHAPS, 0.04% bromophenol blue) and was used to rehydrate 17 cm IPG strips (pH 3-10, non-linear; Bio-Rad) overnight. First dimension isoelectric focusing (IEF) was carried out with IPGphor II (GE Healthcare). The strips were focused at a constant temperature of 20°C with approximately 55 kVh (150 V for 3 hours, gradient to 300 V for 1 minute, 300 V for 5 hours, gradient to 8000 V for 4 hours, 8000 V for 4 hours, gradient to 400 V for 30 minutes, 400 V for 4 hours). The strips then were incubated with equilibration buffer (50 mM Tris-HCL pH 8.8, 6 M urea, 30% glycerol, 2% sodium dodecyl sulfate, 0.02% bromophenol blue) containing 65 mM DTT for 15 minutes, followed by 130 mM iodoacetamide in equilibration buffer for the next 15 minutes. The second dimension SDS-PAGE was performed by mounting the IPG strips onto 8% to 18% polyacrylamide gradient gel (Jule Precast Gels, USA) and running the gels in the Protean II multi cell electrophoresis system (Bio-Rad) at 16 mA/gel for the initial hour and 25 mA/gel at 10°C constantly until bromophenol blue reached the bottom of the gel. Following this, the gels were scanned on a Typhoon Trio variable mode imager (GE Healthcare) at 100 microns resolution to produce a Cy2, Cy3 and Cy5 image for each gel according to the manufacturer's protocol. The images were cropped with ImageQuant software (GE Healthcare) and analyzed by automated Difference In-gel Analysis (DIA) and Biological Variation Analysis (BVA) using Decyder software version 6.5 (GE Healthcare). After the gels were scanned, they were removed from the glass plates fixed with 10% methanol, 7% acetic acid overnight, stained with Coomassie Brilliant Blue G250 (Coomassie Brilliant Blue G250 0.5 g, Ammonium sulphate 50 g, 85% Phosphoric Acid 6 mL in 500 mL water and add 125 mL methanol before use) overnight and de-stained with 1% acetic acid overnight.

### Mass Spectrometry and Protein identification

Spots selected for protein identification were manually excised using a scalpel, de-stained in 60% (v/v) 50 mM ammonium bicarbonate (pH 7.8), 40% (v/v) acetonitrile and then dehydrated in 100% acetonitrile for 1 minute before being dried in a vacuum centrifuge. Gel pieces were rehydrated in 10 μL trypsin solution (12 ng/μl porcine modified sequencing grade trypsin in 50 mM ammonium bicarbonate, pH 7.8) at 4°C for 1 hour. Excess trypsin solution was removed and 15 μL of 50 mM ammonium bicarbonate (pH 7.8) was added prior to incubating overnight at 37°C. For MALDI-TOF MS analysis, 1 μL of peptide was spotted onto a target plate with an equal volume of matrix solution (10 mg/mL α-cyano-4-hydroxycinnamic acid in 70% (v/v) acetonitrile, 1% (v/v) TFA). Mass spectra were acquired in the mass:charge range of 875-3500 m/z on a QSTAR XL mass spectrometer equipped with an oMALDI source (Applied Biosystems Inc., Foster City, CA, USA). The monoisotopic peak masses were subjected to database searching against the MSDB comprehensive non-redundant database using MASCOT vr 2.0 (Matrix Science, London, UK). Parameters for protein identification included searching with a mass error tolerance of 50 ppm per peptide, 1 missed tryptic cleavage, and allowing oxidation of methionine as an optional modification. Confident matches were defined by the MASCOT score and statistical significance (p < 0.05), the number of matching peptides and the percentage of total amino acid sequence covered by those matching peptides. Peptide mixtures that provided poor initial mass spectra were concentrated and desalted using C18 PerfectPure reverse-phase micro-columns (Eppendorf, Düsseldorf, Germany) according to the manufacturer's instructions and eluted in matrix solution directly onto the target plate. MALDI-TOF MS was then performed as described above.

### Functional analysis of protein findings

Once identified with mass spectrometry, the gene encoding counterpart of each individual protein was searched in the Swiss-prot database http://www.expasy.org based on their Swiss-prot database accession numbers. Afterwards, the gene symbol and the fold change of each protein were loaded to Metacore http://portal.genego.com and DAVID http://david.abcc.ncifcrf.gov/ to determine the pathways and biological processes associated with each individual protein. The analysis was performed using the algorithm within the software. The pathways and processes statistically significant to the data set were represented by maps in the database and network, respectively.

### Functional Validation of proteins using Western blotting and Immuno-histochemistry

Western blot and immunohistochemistry were employed to validate the 2D-Dige data. A subset of the same samples for the 2D-Dige study was used for the western blot. 40 ug of proteins were separated by 12% SDS-PAGE and then transferred to PVDF membranes (Millipore, USA) or nitrocellulose membranes (Amersham, USA) using Bio-Rad apparatus (Bio-Rad, USA). Membranes were blocked in 5% skim milk powder or 5% BSA in Tris-buffered saline (TBS) (20 mM Tris and 0.9% NaCl, pH 7.4) for 1 hour at room temperature. Following that, they were incubated for 2 hours at room temperature with each of the following primary antibodies: rabbit anti-Carbonic Anhydrase II (1:10000), Creatine kinase MT (1:75), Glutamine Synthetase (1:2000) (Abcam, US) and with another primary antibody rabbit anti-CRMP2 (1:4000) (Abcam, US) incubated overnight at 4°C. Mouse anti-Actin (1:6000, DAKO, USA) was used as control antibody. Membranes were washed four times with TTBS (TBS with 0.05% Tween20) and then incubated for 1 hour with anti-rabbit HRP-conjugated secondary antibody (Dako, USA; 1:6000) followed by chemiluminescence ECL detection (GE, USA) and exposure to autoradiography film (Kodak, France). Films were scanned with HP scanjet8200 (HP, USA) and the images were collected and analysed using Imagel software http://rsbweb.nih.gov/ij/. Statistically significant differences between patients were estimated with the Mann-Whitney test (P-value < 0.05).

Different samples were used for immuno-histochemistry. The serial sections of frontal lobes from 1 HAD versus 1 HIV non-dementia patients were used for the experiment. Patient details were well documented previously (Patient A and D in [99]). Tissues were fixed in 20% formalin, followed by paraffin embedding. Sections were cut into 6 μm thick for immuno-histochemical staining. Heat antigen retrieval was performed using citrate buffer (pH 6.0) or EDTA buffer (pH 8.0). 3% H2O2, NH4Cl, Glycine, goat serum and Bovine Serum Albumin (BSA) (Aurion) blocking steps were used. Slides then were incubated over night at 4°C or at room temperature with the same primary antibodies used for western blot: rabbit anti-Carbonic Anhydrase II (1:800), Creatine kinase MT (1:10), Glutamine Synthetase (1:100) and rabbit anti-CRMP2 (1:100) (Abcam, US), washed in PBS and then incubated for one hour with the swine anti-rabit HRP-conjugated secondary antibody (1:100; Dako, USA). The detection were made using DAB kit (Dako, USA). The images were captured using a Leica microscope and analysed neuropathologically.

## Competing interests

The authors declare that they have no competing interests.

## Authors' contributions

LZ fully performed the work, analyzed data and drafted the paper. ED assisted with protein extraction and isolation. BC assisted with the Mass Spectrometry and protein annotation identification. RR participated in the protein extraction and quantitation. BW and AK participated in the data analysis. BJB participated in finalizing clinical diagnosis criteria and drafting of the clinical aspects of the paper. ST and HR provided full assistance with the validation of proteins by Western blot. TN contributed to immunohistochemical results interpretation and analysis. KG assisted with proteomic analysis. NKS conceived, designed and coordinated, along with providing assistance with drafting the manuscript. All authors read and approved the final manuscript.

## Supplementary Material

Additional file 1 **Gene Ontology cellular locations of the identified proteins**. Additional file 1 shows the bar chart of Gene Ontology cellular locations of the identified proteins. The x-axis is the log (p value) and the y-axis is the rank of all the significant locations. On the right, a detailed description for them is shown. Click here for file

Additional file 2 **Gene Ontology molecular functions of the identified proteins**. Additional file 2 shows the bar chart of Gene Ontology molecular functions of the identified proteins. The x-axis is the log (pValue) and the y-axis is the rank of all the significant molecular functions. On the right, a detailed description for them is shown. Click here for file

Additional file 3 **Gene Ontology processes of the identified proteins**. Additional file 3 shows the bar chart of Gene Ontology processes of the identified proteins. The x-axis is the log (p-value) and the y-axis is the rank of all the significant biological processes. On the right, the text describes them in details. Click here for file

Additional file 4 **Clinical files of all patients**. Click here for file
