# Supplementary figures and images for: First evidence of overlaps between HIV-Associated Dementia (HAD) and non-viral neurodegenerative diseases: proteomic analysis of the frontal cortex from HIV+ patients with and without dementia
Source: Mol Neurodegener. 2010 Jun 24;5:27. doi: 10.1186/1750-1326-5-27 (PMC2904315; doi:10.1186/1750-1326-5-27)

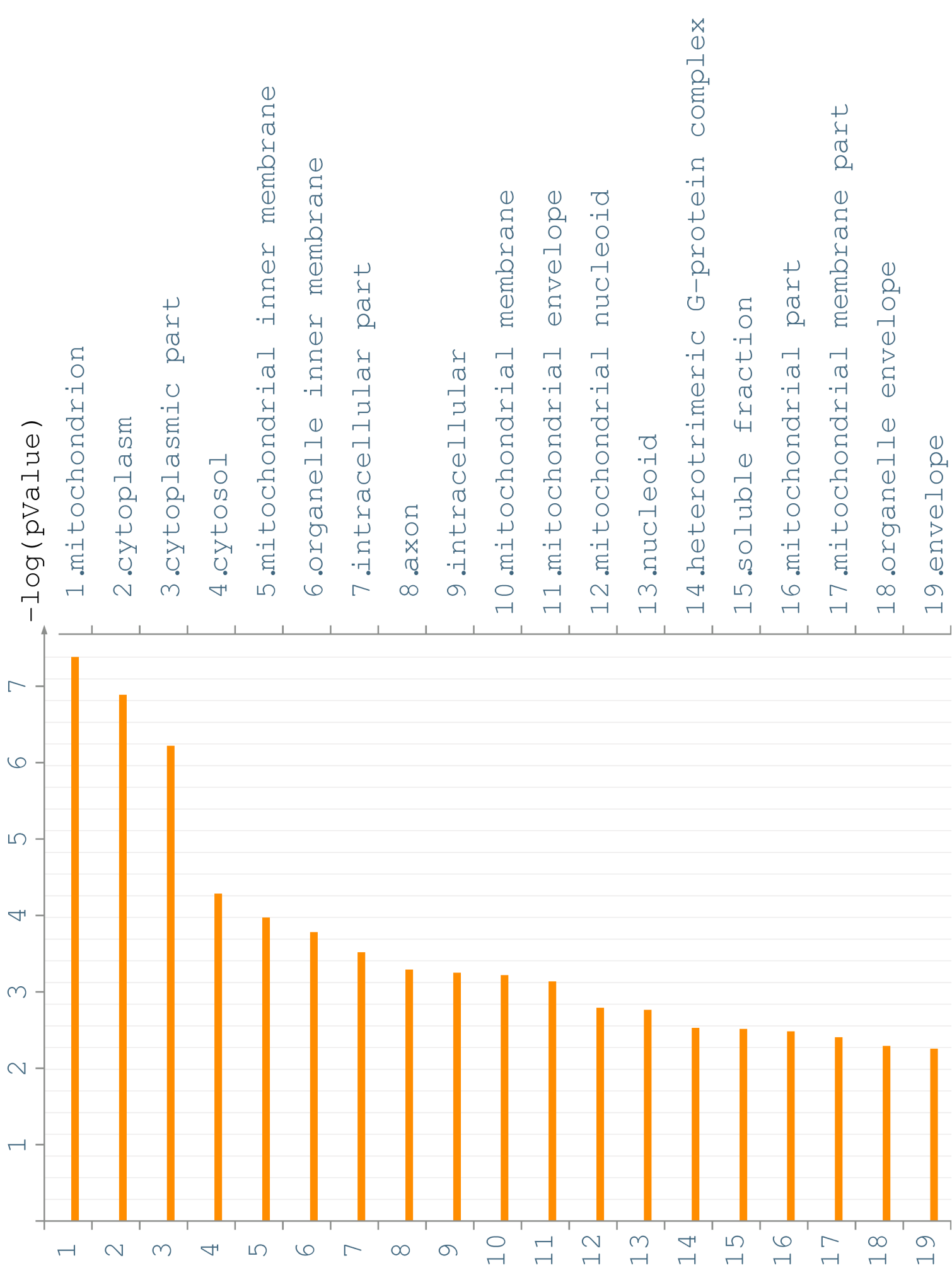

Supplement: Additional file 1 — Gene Ontology cellular locations of the identified proteins. Additional file 1 shows the bar chart of Gene Ontology cellular locations of the identified proteins. The x-axis is the log (p value) and the y-axis is the rank of all the significant locations. On the right, a detailed description for them is shown. [file 1750-1326-5-27-S1.pdf]

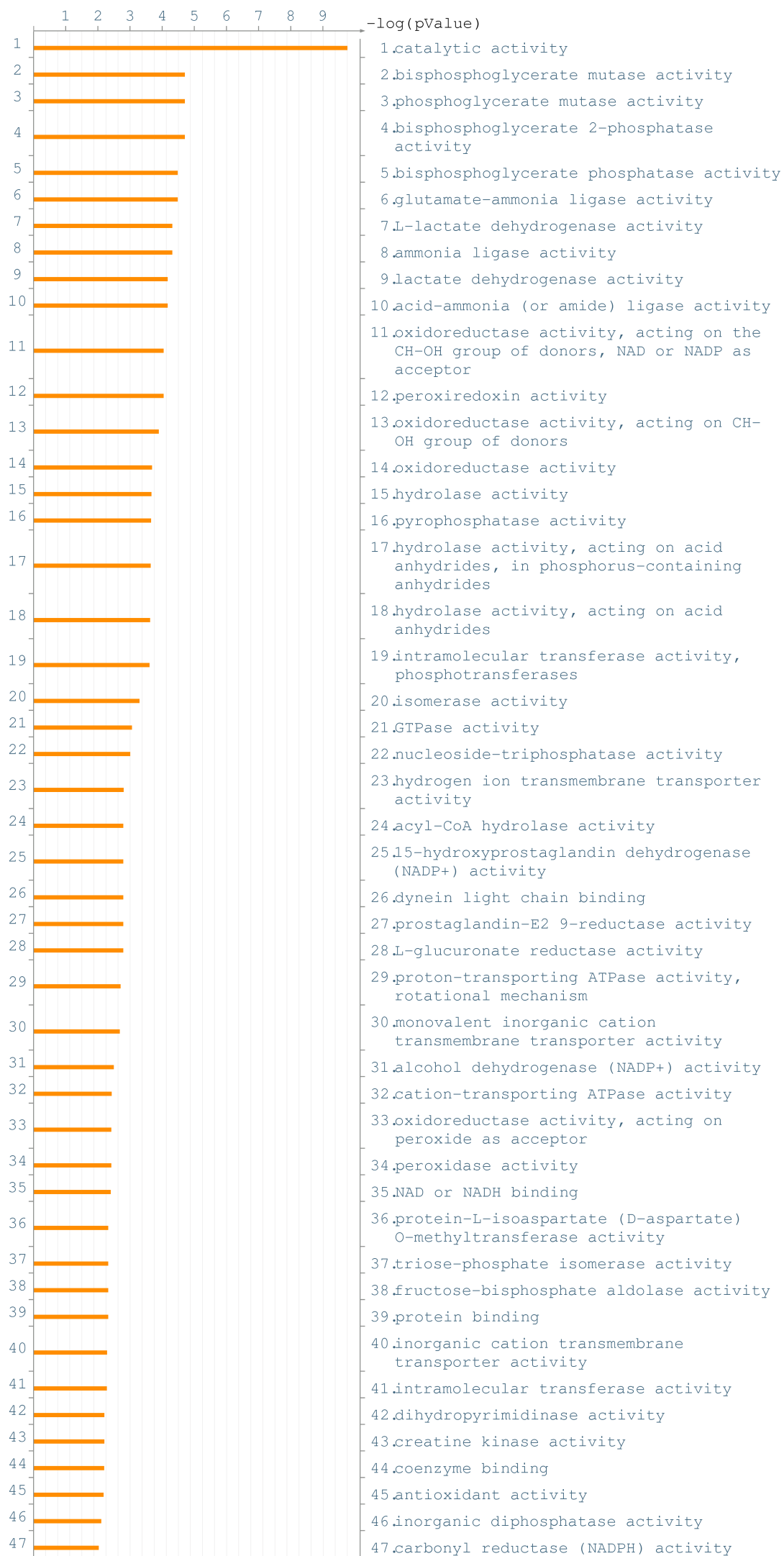

Supplement: Additional file 2 — Gene Ontology molecular functions of the identified proteins. Additional file 2 shows the bar chart of Gene Ontology molecular functions of the identified proteins. The x-axis is the log (pValue) and the y-axis is the rank of all the significant molecular functions. On the right, a detailed description for them is shown. [file 1750-1326-5-27-S2.pdf]

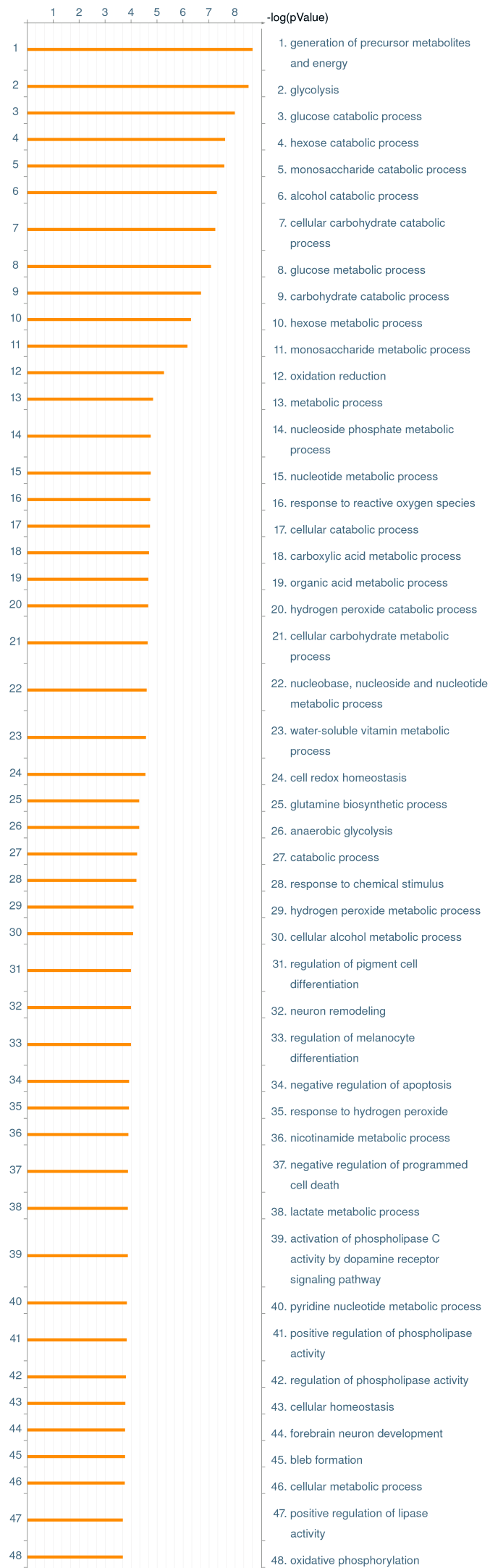

Supplement: Additional file 3 — Gene Ontology processes of the identified proteins. Additional file 3 shows the bar chart of Gene Ontology processes of the identified proteins. The x-axis is the log (p-value) and the y-axis is the rank of all the significant biological processes. On the right, the text describes them in details. [file 1750-1326-5-27-S3.pdf]
