# Supplementary material for: First evidence of overlaps between HIV-Associated Dementia (HAD) and non-viral neurodegenerative diseases: proteomic analysis of the frontal cortex from HIV+ patients with and without dementia
Source: Mol Neurodegener. 2010 Jun 24;5:27. doi: 10.1186/1750-1326-5-27 (PMC2904315; doi:10.1186/1750-1326-5-27)
Supplement: Additional file 4 — Clinical files of all patients. [file 1750-1326-5-27-S4.pdf]

Group[a]	Sample ID	Labeling dye	Sex[b]	Age at death	ART/ARV	Non-HIVE neuropathology	Duration from seropositive for HIV to death(year)
HAD	7766	Cy5	M	38	N/A	N/A	12
	2057	Cy3	M	45			
	2453	Cy3	M	63			
	MW	Cy5	M	23	AZT,DDI		3/4
	1121	Cy5	M	42	N/A	Leukoencephalopathy	19
	2434	Cy3	M	62			
	7659	Cy5	M	32		N/A	9
	Gc16	Cy3	M	U			
	H0011gm	Cy3	M	U			
HAnD	2012	Cy5	M	49	NVP, NFV, ZDV, SQV, D4T, 3TC, FTV, DDI, RTV, NVP, ABC, CBV, APV, TZV, KTA	Minimal non-diagnostic abnormalities	10
	CA303	Cy3	M	67		N/A	
	CC163	Cy3	M	45	3TC, ABC, KTA, NFV, RTV, TFV	N/A	21
	Ca247	Cy5	M	55	3TC, DLV, TFV, NFV	N/A	11
	H0011db	Cy5	M	35			
Internal control		Cy2					

[a] HAD: HIV associated dementia; HAnD: HIV non-dementia

[b] M, male; F, female

N/A: no data available

AZT: zidovudine, ddI: didanosine, NVP: nevirapine, NFV: nelfinavir, ZDV: zidovudine, SQV: saquinavir, D4T: stavudine, 3TC: 2'-deoxy-3'-thiacytidine, FTV: fortovase, RTV: ritonavir, ABC: abacavir, CBV: combivir, APV: amprenavir, TZV: trizivir, TFV: tenofovir, DLV: delavirdine
